# Failure mode and effects analysis outputs: are they valid?

**DOI:** 10.1186/1472-6963-12-150

**Published:** 2012-06-10

**Authors:** Nada Atef Shebl, Bryony Dean Franklin, Nick Barber

**Affiliations:** 1 Department of Practice and Policy, UCL School of Pharmacy, BMA House, Mezzanine Floor, Tavistock Square, London, WC1H 9JP, UK; 2 Department of Pharmacy Practice, The School of Pharmacy, University of Hertfordshire, Hatfield, AL10 9AB, UK; 3 Centre for Medication Safety and Service Quality, Pharmacy Department, Imperial College Healthcare NHS Trust and UCL School of Pharmacy, London, UK; 4 Pharmacy Department, Charing Cross Hospital, Fulham Palace Road, London, W6 8RF, UK

## Abstract

**Background:**

Failure Mode and Effects Analysis (FMEA) is a prospective risk assessment tool that has been widely used within the aerospace and automotive industries and has been utilised within healthcare since the early 1990s. The aim of this study was to explore the validity of FMEA outputs within a hospital setting in the United Kingdom.

**Methods:**

Two multidisciplinary teams each conducted an FMEA for the use of vancomycin and gentamicin. Four different validity tests were conducted:

· Face validity: by comparing the FMEA participants’ mapped processes with observational work.

· Content validity: by presenting the FMEA findings to other healthcare professionals.

· Criterion validity: by comparing the FMEA findings with data reported on the trust’s incident report database.

· Construct validity: by exploring the relevant mathematical theories involved in calculating the FMEA risk priority number.

**Results:**

Face validity was positive as the researcher documented the same processes of care as mapped by the FMEA participants. However, other healthcare professionals identified potential failures missed by the FMEA teams. Furthermore, the FMEA groups failed to include failures related to omitted doses; yet these were the failures most commonly reported in the trust’s incident database. Calculating the RPN by multiplying severity, probability and detectability scores was deemed invalid because it is based on calculations that breach the mathematical properties of the scales used.

**Conclusion:**

There are significant methodological challenges in validating FMEA. It is a useful tool to aid multidisciplinary groups in mapping and understanding a process of care; however, the results of our study cast doubt on its validity. FMEA teams are likely to need different sources of information, besides their personal experience and knowledge, to identify potential failures. As for FMEA’s methodology for scoring failures, there were discrepancies between the teams’ estimates and similar incidents reported on the trust’s incident database. Furthermore, the concept of multiplying ordinal scales to prioritise failures is mathematically flawed. Until FMEA’s validity is further explored, healthcare organisations should not solely depend on their FMEA results to prioritise patient safety issues.

## Background

There has been growing awareness that proactive or prospective analysis methods, such as those that have been used in other high hazard industries, provide additional benefits for improving quality and safety in healthcare [1]. In the last few years, the most prominent proactive risk assessment technique used within healthcare has been Failure Mode and Effects Analysis (FMEA).

FMEA is a prospective risk assessment tool designed to promote patient safety by mapping out the process of care, then identifying potential failures that may occur in this process, in order to understand how and why errors or failures occur. The FMEA process and steps are briefly described in Table 1.

**Table 1:** **FMEA steps**[2-4]

**STEP**	**Comments**
1- Defining the Topic	The topic is usually a high-risk process
2- Assembling the Team	An FMEA team should be multidisciplinary.
3- Graphically describing the process using flowcharts	Identify the failures that can occur, their causes and effects.
4 -Calculating the risk priority number (RPN). The RPN is calculated for each failure identified in step 3	This is done by multiplying the severity, probability and detectability scores (usually using 10-point scales, accompanied by written descriptions for numerical scores.) Severity relates to the seriousness of the injury or impact that could ultimately result if a failure occurs. The probability of occurrence is the likelihood that something will happen. Detectability is the degree to which something can be discovered or noticed, i.e. if this failure occurs, how likely is it to be detected?
5- Actions and outcome measures	The team makes recommendations to decrease or eliminate the risk of failures.

Sensitivity to small changes: Small variations in one scoring scale can lead to very different effects on RPN, depending on the values of other factors. For example (Table 2):

FMEA’s use in healthcare has been established during the last decade, particularly in the USA, and has been endorsed by a number of patient safety agencies such as the Joint Commission, Institute for Healthcare Improvement (IHI) and the Institute for Safe Medication Practices (ISMP). In the recent years, FMEA’s reliability has been questioned and explored [5-7]; while the validity of its use in healthcare has been questioned but not yet assessed [7]. In this study, we wished to explore the characteristics of FMEA by studying its validity.

Validity is concerned with the accuracy of data [8]; it is an assessment of whether an instrument measures what it aims to measure [9]. In science, validity is essential to a research proposal’s theoretical framework, design and methodology, including how well specific tools or instruments measure what they are intended to measure [10].

The aim of this study was to explore the validity of the FMEA outputs, and where appropriate the tool itself, by four different methods including:

1. Face validity: Refers to the investigators’ or an expert panel’s subjective assessment of the presentation and relevance of the tool in question [9].

2. Content validity: Involves the judgment, usually by an expert panel, about the extent to which the contents of the FMEA results appear to examine and include the domains it is intended to measure [9].

3. Criterion validity: Refers to the extent to which the method correlates with other measures of the same variable [11]. To demonstrate criterion validity, the results are compared with established standard methods of collecting the same information.

4. Construct validity: Carmines and Zeller [12] report that construct validity is concerned with the extent to which a particular measure relates to other measures consistent with theoretically derived hypotheses concerning the concepts that are being measured, i.e. the validity seeks agreement between a theoretical concept and a specific measuring procedure or device.

## Methods

In 2009, two multidisciplinary groups were recruited from 2 large teaching hospitals within the same English National Health Service (NHS) Trust, to conduct separate FMEAs in parallel on the prescribing, administering and monitoring of vancomycin and gentamicin. The groups followed the standard FMEA steps in Table 1. Results of these two FMEAs have been published in detail elsewhere [5].

Following the completion of these two FMEAs, the validity of the FMEA’s output was explored in the same two hospitals where the FMEA meetings took place [5]. Ethical approval was granted by the local Research Ethics Committee before the start of the study.

The above validity tests were applied in the present study as follows:

1. Face validity: This was taken to refer to the researchers’ subjective assessment of the process of vancomycin and gentamicin use, as mapped out by the FMEA teams.

2. Content validity: This involved the judgment of healthcare professionals who did not participate in the FMEA teams who determined the extent to which the outputs of the FMEA appeared to include all the domains judged to be required.

3. Criterion validity: This involved assessing the extent to which outputs of the vancomycin and gentamicin FMEA correlated with other similar objective measures.

4. Construct validity: A key theory underpinning FMEA is to prioritise failures, and this is achieved by calculating the RPN value. The mathematical properties of the scoring scales used were therefore assessed and their use in FMEA evaluated.

The first three tests explored the validity of the FMEA’s ouputs, while construct validity related to the FMEA tool itself.

### Face validity

To explore the face validity of the FMEA outputs, observational work was carried out. A researcher (N.S) shadowed a number of pharmacists on their daily clinical pharmacy visits to medical and surgical wards for a period of two weeks to observe use of vancomycin and gentamicin in practice. Two days were also spent in the microbiology and chemistry laboratories. Consultant ward rounds were attended, and nurses observed preparing and administering vancomycin and gentamicin.

Other aspects of the process such as blood sampling from patients, nurses receiving laboratory results on the phone or doctors checking the computer systems for the results of drug assays were not directly observed as they occurred at unpredictable times during the day. Instead, information about these steps was obtained through conversations with the ward nurses and pharmacists. The process maps created by the two FMEA teams were then compared with the researcher’s observations.

### Content validity

Initially 70 healthcare professionals, comprising senior doctors, junior doctors, pharmacists, nurses, laboratory personnel, service managers and risk managers had been invited to participate in the FMEA meetings but only 14 actually participated [5]. The remaining 56 were contacted again after the FMEA was completed and shown the FMEA flow chart and the potential failures identified. They were invited to comment as to whether or not they agreed with the mapped process and the potential failures identified. E-mail reminders were sent once a week for three weeks.

### Criterion validity

Incidents involving the use of intravenous (IV) gentamicin or vancomycin were retrieved from the trust’s incident report database for the period January 2006 to January 2009. Incidents that did not specifically mention vancomycin or gentamicin or were related to the use of these antibiotics in children and patients on dialysis were excluded. Incidents involving the continuous infusion of vancomycin, for example in the intensive care unit, were also excluded because this dosing regimen had been excluded from the FMEA discussions.

Criterion validity was then explored using two approaches. First, the failures identified by the FMEA teams were listed together with their FMEA probability scores. The corresponding incidents reported on the hospital’s incident database were then listed. The reported frequency of incidents similar to the FMEA failures was determined for the three year period.

Second, the severity and the probability of recurrence of the incidents reported on the database, as assessed by the healthcare professionals reporting the incidents, and the severity and probability scores of their corresponding FMEA failures were tested for correlation using Spearman’s correlation.

### Construct validity

As the main theory behind FMEA is to prioritise failures, and this is achieved by calculating the RPN value, the mathematical properties of the scoring scales used were explored and their use in FMEA evaluated.

## Results

### Face validity

To assess the face validity of FMEA, a flowchart for prescribing, administering and monitoring vancomycin and gentamicin developed by the researcher through observations was compared to the mapped processes prepared by the FMEA teams [5]. The flowchart style developed by the researcher included ‘yes’ and ‘no’ choices, while the FMEA teams developed a simple event line and included sub processes under each main step identified. Other than this, the main difference between the flowcharts mapped by the FMEA teams and that developed by the researcher was the level of detail presented in the FMEA flowchart. The teams identified more detailed sub processes to help them list the failures more easily. In spite of these differences, the main steps identified by both FMEA teams were the same as those identified by the researcher through observations on the ward. These steps included prescribing, administering and monitoring the use of vancomycin or gentamicin. Key issues such as microbiological cultures and sensitivities, empirical treatment and modifying treatment after levels reported were also acknowledged by both FMEA teams and by the researcher.

### Content validity

Only four (7.5%) of the 56 healthcare professionals agreed to comment about the FMEA, and only three (5.4%) actually replied after three weeks of E-mail reminders. All three respondents were medical consultants.

The consultants who reviewed the FMEA had a number of comments and additions to the completed FMEA sheet. The first consultant did not have comments regarding the FMEA results but instead questioned the evidence behind the use of the scoring scales. On the other hand, the second consultant commented on the sub processes and failure identified, indicating that there were important failures that the groups did not identify such as checking for allergies and recording the patient’s weight and age. Furthermore, this consultant disagreed with some of the RPN scorings for some of the failures and stated that some failures deserved a much higher priority than others. Therefore, from the consultant’s point of view, the RPN values of some failures may have differed if she/he had participated in the FMEA meetings. The third consultant was content with the FMEA data provided and did not make any further comments.

### Criterion validity

In total, 52 incidents concerning vancomycin or gentamicin were retrieved for the period January 2006 to January 2009 but only 22 met the inclusion criteria for analysis. Thirteen of the 22 (59%) reported incidents were of types that had been identified by the FMEA teams and were therefore compared to the FMEA failures. Of the remaining nine incidents the FMEA teams had not identified, seven (78%) were related to omitted doses and two (22%) reported that the wrong route for the medication had been prescribed on the drug chart, neither of which were failures identified by the FMEA teams.

**Figure 1:**
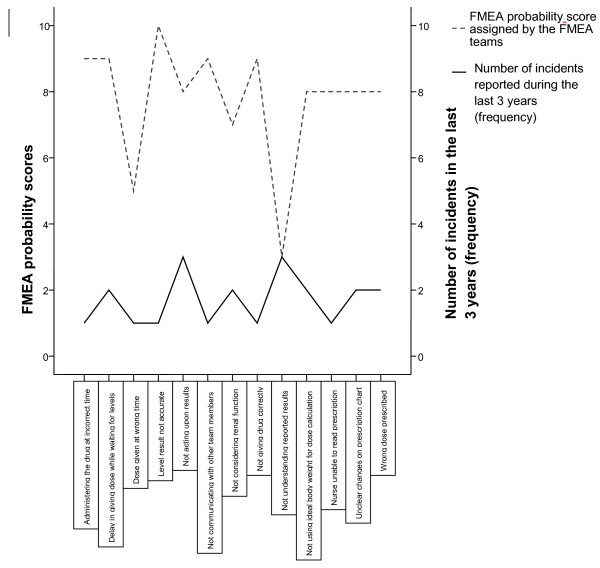
Comparing FMEA probability scores.

First the frequency of incident reporting was compared to the probability scores assigned by the FMEA teams to the same failures. There seemed to be little relationship between how frequently failures were reported in comparison to how often the team perceived that these failures occurred (Figure 1).

**Figure 2:**
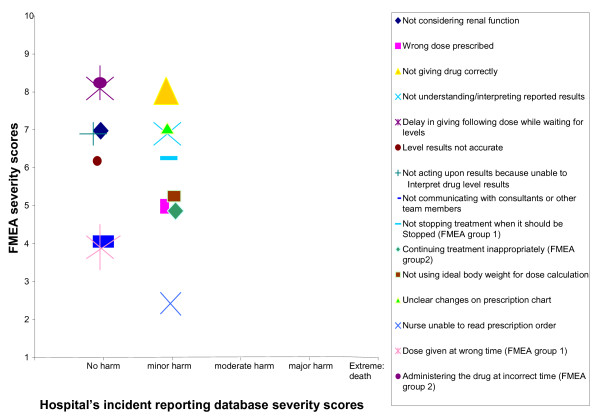
Severity scores for incidents reported on the organisation’s incident reporting database and the equivalent FMEA failures and their severity scores.

Secondly, the severity and the probability of recurrence of the incidents reported on the database (as assessed by the healthcare professionals reporting the incidents), and the severity and probability scores of the corresponding FMEA failures were compared. Figure 2 shows that the severity scores on the trust’s incident reporting database were either ‘no harm’ or ‘minor harm’, while for the FMEA failures, the lowest severity score was two (for one failure) and the highest score was an eight (= major injury; for three failures), with the majority of scores ranging between five and seven.

**Figure 3:**
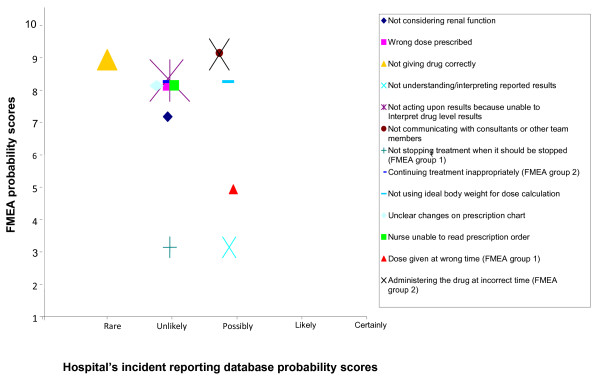
Probability scores for incidents reported on the organisation’s incident reporting database and the equivalent FMEA failures and their severity scores.

As for the probability scores, Figure 3 shows that the lowest probability score for the FMEA failures was three, for two failures. All the remaining scores ranged between five (one occurrence every six months) and nine (one occurrence every three to four days); and the only failure given a probability of nine by the FMEA team had an equivalent probability of ‘rare’ reported on trust’s incident reporting database. Overall, the FMEA participants anticipated that the majority of failures would occur again at least once a month (probability score of eight), while the majority of similar incidents reported on trust’s incident reporting database were considered unlikely to recur, i.e expected to occur again annually.

The correlation between the FMEA severity and probability scores, and the trust’s incident reporting database severity and probability scores was not significant (r_s_: 0.42 and r_s_: 0.77 respectively).

### Construct validity

The RPN is calculated by multiplying three ordinal scales: severity scores, probability scores and the detectability scores. In an ordinal scale, the categories have an ordered or ranked relationship relative to each other; however, the amount of difference between ranks is not specified. Mathematically, ordinal scales incorporate the relationships of equivalence (=), ‘greater than (>)’ and ‘less than (<)’ [13]. For example, a doctor might use a scale of 1–5 to indicate degree of improvement in some condition, from 1 (no improvement) to 5 (complete cure). While we know that a score of 4 is better than a score of 2, there is no implication that 4 is ‘twice as good’ as 2. Nor is the improvement from 2 to 4 necessarily the same “amount” of improvement as the improvement from, say, 1 to 3. All we know is that there are 5 categories, with 2 being better than 1 and 3 being better than 2.

Siegel [13] states that the intervals of an ordinal scale are not identical to each other (as intervals on an ordinal scale are determined subjectively and will differ with each user), and thus ordinal scales cannot be subjected to the numerical system known as arithmetic; in other words ordinal numbers cannot meaningfully be multiplied or divided [14]. In FMEA however, the ordinal scales of severity, probability and detectability are multiplied to produce the RPN, which breaches the mathematical properties of the ordinal scales. Bowles [14] highlights four main limitations of using the RPN in the way that it is currently used in FMEA:

1. Holes in the scale: Many of the numbers in the range of 1 to 1000 (assuming 10-point scoring scales are used) cannot be formed from the product of severity, probability and detectability. While it is true that the numbers cover a range from 1 to 1000, 88% of that range is empty with the first four possible RPN values covering 0.4% of the scale, while the last four values cover 20% of the scale. No number having a prime factor greater than 10 can be formed. Thus the numbers 11, 22, 33 or even 990, which are all multiples of 11 cannot be formed and are excluded. 1000 is the largest number, but 900 is the second largest followed by 810, 800, 729 and 720. In this case, can we say that the difference between 810 and 900 is the same or less than the difference between 900 and 1000?

2. Duplicate RPN values: Since 1000 numbers are produced from the product of severity, probability and detectability but only 120 of them are unique, the majority of the RPN values can be formed by several ways. For example, the RPN values of 60, 72 and 120 can each be formed from 24 different combinations of severity, probability and detectability scores.Although the RPN values may be identical, their risk implications may be different.

3. 

**Table 2:** Example of RPN’s sensitivity to small changes

Severity	Probability	Detectability	RPN
3	8	8	192
8	3	8	192

A 1 point change in the severity in the first example causes a 64 point change in the RPN, whereas in the second a 1 point change in severity causes only a 24 point change (Table 3).

**Table 3:** Example of RPN’s sensitivity to small changes

Severity	Probability	Detectability	RPN
4	8	8	256
9	3	8	216

4. Comparing the RPNs: Bowles [14] also argues that comparing the RPN values is generally not possible without incorporating some costs that quantify how the reductions along one scoring scale (for example the severity scores) relate to changes along the other scoring scales (probability and detectability scores).

## Discussion

In this study the validity of FMEA was explored by assessing different types of validity for the FMEA. Four different types of validity were assessed: face, content, criterion and construct validity. No previous work has formally explored the validity of FMEA outputs.

Face validity of the FMEA mapped outputs was positive as both groups included the main steps identified by the researcher through observations. Content validity of the FMEA was explored by presenting the FMEA findings to other healthcare professionals. These healthcare professionals identified other potential failures within the process of vancomycin and gentamicin use. Furthermore, the FMEA groups failed to include failures related to omitted doses, yet these were the failures most commonly reported in the trust’s incidents database. Testing criterion validity of the FMEA was done by comparing the FMEA findings with data reported on the trust’s incident report database. The results showed no significant correlation between the scores reported by the FMEA team and those reported on the trust’s incidents database as the FMEA team scored their severity and probability scores much higher than those on database. Finally construct validity was assessed by exploring the relevant mathematical theories involved in calculating the risk priority number (RPN). Construct validity of FMEA has been previously addressed in a number of engineering articles, where the use of RPN has been criticized [14,15]. These papers assessed construct validity indirectly by exploring the relevant mathematical theories involved in calculating the RPN, which proved to be flawed because the RPN calculations are based on invalid mathematical calculations that breach the properties of the scales used.

The study’s results indicate that the FMEA team must be multidisciplinary in order to identify as many potential failures as possible; and yet the teams may need to acknowledge that they might not be able to capture all expected failures. As for criterion validity, FMEA participants had the tendency to overestimate the severity of the effect of the failure in comparison to those reporting the incidents on the database. This is perhaps due to the fact that with the incident reporting database, the error or failure is reported retrospectively; the healthcare professional reporting the incident has witnessed the effect of the error, if any, on the patient and thus the reported severity score is based on the actual effect of the error on the patient. While with FMEA, the failures identified by the groups are identified as prospective failures, i.e. potential failures. This perhaps made it difficult for the FMEA team members to predict the likely effect of this failure and thus in some cases the groups were perhaps presuming the worst effects of certain failures on the patients concerned.

### Why is validity important?

Kirwan [16] reported that lack of evidence of validity leads to two basic problems when using techniques such as FMEA: firstly, there is scepticism as to whether the techniques available have any empirical predictive validity, and secondly, technique developers and assessors get little useful feedback on how to improve the technique’s predictive accuracy and precision. Furthermore, relying on invalid results to improve any process may lead to unnecessary or inappropriate costly changes within an organisation. Validation, therefore, is essential as a general quality assurance process and to generate the ability to fine-tune risk assessment techniques. Kirwan [16] further explained that techniques that depend on significant judgment either by assessors or experts may fail to accurately quantify the risks. Therefore it is necessary that objective tests are carried out to ensure validity of these tools, thereby checking and improving the accuracy of the risk assessment as a whole [17].

The purpose of FMEA is to estimate the risk of potential failures and prioritise the failures that require the most attention, whether this is because they are assumed to be the most severe, the most probable or the least detectable failures or a combination of these. Thus if patient safety becomes reliant on such a technique then it is essential to ensure the results produced are consistent and accurate, irrespective of the team using the tool, especially since FMEA entails a lot of time, effort and resources.

### Healthcare FMEA (HFMEA)

In 2001, the USA’s Veteran’s Administration (VA) National Centre for Patient Safety (NCPS) [3] specifically designed the Healthcare FMEA (HFMEA) tool for risk assessment in the healthcare field. HFMEA and FMEA have similarities at their core, but deal with detectability differently, and HFMEA uses 4-point scales instead of 10-point. They both involve the same 5 basic steps (Table 1). The main difference between them lies in the scoring step; HFMEA detectability scores are only determined if the failure identified warrants further action, as determined by a decision tree.

Although HFMEA was not formally evaluated in this study, it is assumed that at least some of the problems inherent within FMEA will also be present in HFMEA and will also affect the reliability and validity of HFMEA’s results.

### Limitations

There were two main limitations to our work: First, when exploring the content validity only three consultants were able to provide feedback on the completed FMEA although reminders to all 56 potential respondents were sent out each week for three consecutive weeks. The low response rate could potentially be attributed to two main issues; either healthcare providers contacted may never have heard about FMEA and thus were not interested to ‘learn’ about a new tool and then criticise it, or they were familiar with FMEA but it was perceived as being too time consuming for them to go through the entire FMEA worksheet and make comments. Second, comparing the FMEA failures with incidents reported on the trust’s reporting database proved to be a challenge because reporting databases are known to capture a small proportion of incidents. In a recent comparison between reporting systems and systematic review of records, the reporting systems detected only about 6% of the adverse events found by systematic review of records [18]. Nurses estimate that only between 25% and 63% of medication errors are actually reported [19,20]; while a study by Franklin et al. [21] found that spontaneous reporting identified only 1% of all prescribing errors.

### Implications

Improving the content validity of the FMEA outputs maybe achieved by allowing the FMEA teams to use other sources, besides their experience and knowledge, such as hospital audits or incident report databases, to list as many potential failures as possible. However, the main limitation of FMEA would still be the use of numerical values to subjectively rank these potential failures. In addition to this, it would be impractical, expensive and very time consuming to collect objective data for each potential failure identified. Thus the appeal of FMEA as a simple prospective generic tool would not be realised.

In addition to this, when healthcare organisations decide to conduct an FMEA, participating teams must be aware that the conclusions of FMEA are usually short lived, particularly in healthcare. As new evidence-based medicine continues to evolve and guidelines and protocols continue to be periodically updated, along with the introduction of new technologies such as electronic prescribing, clinical decision support or bar-coding, a given set of FMEA results will only be valid for a limited time period and should therefore be updated regularly. Furthermore, the policy of doctors ‘turnover’ or rotations within different hospitals (as within the National Health System in the UK) should be considered. These doctors might be available to participate in the FMEA discussions but their rotations would mean that they may not be around to implement the new changes or teach them to others and thus the FMEA may need to be repeated.

### Recommendations

Practical recommendations for conducting an FMEA have been extensively published including guidelines about how to choose high risk topics, who should participate in the FMEA meetings, how the meetings should be conducted and even how to reach consensus with the participating team [3,22-24]. Reviews related to the use of FMEA in healthcare have all supported its application in healthcare and have encouraged its use, indicating that the Joint Commission in the USA, as well as several other bodies, promote its use. There is no denying that FMEA is a useful prospective tool that allows healthcare professionals to discuss a process of care as a team. However, the results of this study have indicated that FMEA’s validity is questionable and thus the absolute promotion of its use in healthcare may be inappropriate. As Spath [25] (p.116) has stated:

"‘One of the worst practices used in conducting FMEA projects is to use only FMEA techniques to make a process safer since the FMEA methodology for improving the safety of processes has some known limitations.’"

Following the results of this study and previous reliability studies [2,3] it would not be appropriate to recommend the use of FMEA alone as a tool for preventing patient harm. The benefits of gathering a multidisciplinary team to discuss a process of care are clear; However, organisations do not necessarily need to gather a team with the goal of conducting ‘FMEA’. Identifying potential failures is beneficial as it allows the team to share experiences, yet as they are ‘potential failures’ there is no need to translate these failures into numerical representatives including severity, probability and detectability scores. The scores might be useful to guide the team, but the scores should not become the main focus of the process where the aim of the FMEA becomes reducing the RPN values rather than finding solutions to avoid failures or errors from reaching the patient. Furthermore, focusing the FMEA on reducing the RPN values may result in bias results as participants’ focus shifts from patient safety to lowering numerical values. It is also essential that any changes or improvements should be recommended while keeping in mind the feasibility and costs of their implementation as well the resources and methods of evaluating their benefit or effects on patient safety.

As this is the first study to evaluate the validity of FMEA in healthcare, there is significant potential for future studies to further explore each step of FMEAs validity. Ideally future studies should explore criterion validity of FMEA by collecting relevant objective data and comparing it to those provided by the FMEA team data. In addition to this, because FMEA data and outputs are not standard, the data collected could be tailored for each specific FMEA, and thus the validity of the outputs can be further tested or explored rather than the FMEA process itself.

## Conclusion

There are significant methodological challenges in validating FMEA. It is a useful tool to aid multidisciplinary groups in mapping and understanding a process of care; however, the results of our study cast doubt on its validity. FMEA teams are likely to need different sources of information, besides their personal experiences and knowledge, to identify potential failures. As for FMEA’s methodology for scoring failures, there were discrepancies between the teams’ estimates and similar incidents reported on the trust’s incident database. Furthermore, the concept of multiplying ordinal scales to prioritise failures is mathematically flawed. Until FMEA’s validity is further explored, healthcare organisations should not solely depend on their FMEA results to prioritise patient safety issues.

## Misc

Nada Atef Shebl, Bryony Dean Franklin and Nick Barber contributed equally to this work.

## Competing interests

The authors declare that they have no competing interests.

## Authors’ contribution

NS helped design the study, collected the data, analysed and interpreted the data and drafted the manuscript. NB and BDF helped design the study, reviewed the outputs for face validity, interpreted the data and drafted the manuscript. All authors read and approved the final manuscript.

## Pre-publication history

The pre-publication history for this paper can be accessed here:

http://www.biomedcentral.com/1472-6963/12/150/prepub
